# Variability and accuracy of multiple saliva pepsin measurements in laryngopharyngeal reflux patients

**DOI:** 10.1186/s40463-023-00670-5

**Published:** 2023-10-04

**Authors:** Jerome R. Lechien, Francois Bobin

**Affiliations:** 1 Division of Laryngology and Broncho-Esophagology, Department of Otolaryngology-Head Neck Surgery, EpiCURA Hospital, Baudour, Belgium; 2 Department of Human Anatomy and Experimental Oncology, Faculty of Medicine, UMONS Research Institute for Health Sciences and Technology, University of Mons (UMons), Avenue du Champ de Mars, 6, 7000 Mons, Belgium; 3 Department of Otorhinolaryngology and Head and Neck Surgery, Foch Hospital, School of Medicine, UFR Simone Veil, Université Versailles Saint-Quentin-en-Yvelines (Paris Saclay University), Paris, France; 4 Department of Otorhinolaryngology and Head and Neck Surgery, CHU de Bruxelles, CHU Saint-Pierre, School of Medicine, Université Libre de Bruxelles, Brussels, Belgium; 5 Polyclinique Elsan de Poitiers, Poitiers, France; 6 Research Committee of Young Otolaryngologists of the International Federation of Oto-Rhinolaryngological Societies (YO-IFOS), Paris, France

**Keywords:** Laryngopharyngeal, Reflux, Voice, pH monitoring, Laryngeal, Otolaryngology, Saliva, Pepsin

## Abstract

**Objective:**

To study the variability and diagnostic value of multiple salivary pepsin measurements in the detection of laryngopharyngeal reflux (LPR).

**Methods:**

Patients with LPR symptoms were consecutively recruited from December 2019 to Augustus 2022. Twenty-one asymptomatic individuals completed the study. The diagnostic was confirmed with hypopharyngeal–esophageal impedance-pH monitoring (HEMII-pH). Patients collected three saliva samples during the 24-h testing period. Symptoms and findings were studied with reflux symptom score-12 and reflux sign assessment. Sensitivity, specificity, positive (PPV) and negative (NPV) predictive values of pepsin measurements were calculated considering morning, post-lunch and post-dinner samples. The consistency and relationship between HEMII-pH, pepsin measurements, and clinical features were investigated.

**Results:**

Morning, post-lunch and post-dinner saliva pepsin concentrations were measured in 42 patients. Pepsin measurements were 64.9%, 59.5%, and 59.0% sensitive for morning, post-lunch and post-dinner collections at cutoff ≥ 16 ng/mL. Considering the highest concentration of the three pepsin saliva collections, the accuracy, sensitivity, specificity and PPV were 70.5%, 73.0%; 66.7% and 78.9%, respectively. Morning pepsin measurements reported higher consistency, sensitivity, and specificity than post-dinner and post-lunch pepsin measurements.

**Conclusion:**

The collection of several saliva pepsin samples improves the detection rate of LPR. In case of high clinical LPR suspicion and negative pepsin test, a HEMII-pH study could provide further diagnostic information.

## Introduction

Laryngopharyngeal reflux (LPR) is an inflammatory condition of the upper aerodigestive tract tissues related to direct and indirect effect of gastroduodenal content reflux, which induces morphological changes in the upper aerodigestive tract [1]. The symptoms and findings are related to the deposit of pepsin in laryngopharyngeal mucosa and the development of inflammatory reaction and mucosa injuries [2]. Currently, the LPR diagnosis may be confirmed with the 24-h hypopharyngeal–esophageal multichannel intraluminal impedance-pH monitoring (HEMII-pH), which detects pharyngeal reflux events [3]. HEMII-pH is considered as the gold standard for the LPR diagnosis, but this tool remains costly and inconvenient. HEMII-pH is uncommonly used by otolaryngologists [4, 5]. Over the past 2 decades, saliva pepsin measurement was proposed as a cost-effective and minimal invasive diagnostic tool for LPR [6]. However, studies reported inconsistencies regarding the cutoff, the collection time and the appropriate number of saliva samples for the diagnostic. These inconsistencies may be due to the variability of the saliva pepsin concentration over time [6–10].

The aim of this study was to investigate the variability of pepsin saliva concentration throughout the day and its diagnostic value for detecting laryngopharyngeal reflux.

## Material and methods

### Subjects and setting

Patients with suspected LPR regarding laryngopharyngeal symptoms and findings were consecutively recruited from two European hospitals (Saint-Pierre University Hospital of Brussels (Belgium) and Elsan Polyclinic of Poitiers (France)). The LPR diagnosis was confirmed with 24-h HEMII-pH. Gastrointestinal (GI) endoscopy was proposed to elderly individuals and those with gastroesophageal reflux disease (GERD) complaints. The exclusion criteria included the following: active smoker, alcoholic (> 3 alcohol glasses daily), history of upper respiratory tract infection within the last month, neurological or psychiatric illness, head and neck malignancy, head and neck radiotherapy, active seasonal allergies, intake of inhaled corticosteroids, or asthma.

The local ethics committee approved the study protocol (CHU Saint-Pierre, n°BE076201837630). Patients consented to participate.

### Hypopharyngeal–esophageal multichannel intraluminal impedance-pH monitoring

The catheter placement and tracing analyses were described in a previous publication (Versaflex Z®, Digitrapper pH-Z testing System, Medtronic, Europe) [3]. In sum, the catheter was placed in the morning fasting (8:00 AM) and removed the next day in the morning. The catheter was composed of 8 impedance segments and 2 pH electrodes. The six esophageal impedance segments were placed along the esophagus zones (Z1 to Z6) at 19, 17, 11, 9, 7 and 5 cm above the lower esophageal sphincter (LES). The pharyngeal impedance segments were placed 1 and 2 cm above the upper esophageal sphincter (UES) in the hypopharynx. The pH electrodes were placed 2 cm above LES and 2 cm below UES, respectively. According to recent systematic review on HEMII-pH features in healthy individuals [11], the LPR diagnosis criteria was based on the occurrence of > 1 acid (pH ≤ 4.0) or nonacid (pH > 4.0) hypopharyngeal reflux events (off proton pump inhibitors).

### Saliva pepsin measurements

The pepsin concentration was measured in the saliva samples with Peptest® device (RD Biomed Ltd., Hull, United Kingdom). Patients collected 1 to 5 mL saliva samples in the morning (fasting, after waking) and 2 h after the lunch and the dinner. The saliva was collected during the 24-h HEMII-pH period. The saliva was collected into a 30-mL universal sample collection tube containing a pre-established concentration of citric acid. Pepsin is active at acidic pH (around 2.0 to 4.0). The citric acid in the collection tube helps to maintain this low pH level acting as a buffer. It prevents the pH of the sample of increasing, which would deactivate the pepsin and compromise the accuracy of the test results.

The pepsin sample collections were stored in the refrigerator for a period of up to one week, which was found to have no significant impact on the pepsin measurement [12]. A trained lab technician analyzed the samples according to a standardized procedure [12]. The result of the pepsin test was validated when a blue line appeared under the letter C (control) of the device 15 min after applying the sample. The apparition of a blue line under the letter T (test) meant that the pepsin test was positive. The Cube Reader® was used to precisely measure the level of pepsin, the concentration ranging from 1 to 500 ng/mL.

### Clinical outcomes and control group

Demographic outcomes (e.g. age, gender, body mass index) were collected from the patient’s medical record. Reflux Symptom Score-12 (RSS-12) [13] was used to rate the severity and the frequency of symptoms. A RSS-12 > 11 was suggestive of LPR. Findings were evaluated with Reflux Sign Assessment (RSA) [14], which rates oral, pharyngeal and laryngeal signs associated with LPR. An RSA > 14 may be suggestive of LPR.

RSS-12 and RSA were used to recruit 21 asymptomatic individuals who reported RSS-12 < 11 and RSA < 14. Exclusion criteria of asymptomatic individuals were similar to those of LPR patients. They similarly collected saliva samples to measure pepsin.

### Statistical methods

Statistical Package for the Social Sciences for Windows (SPSS version 27.0; IBM Corp, Armonk, NY, USA) was used for the statistical analyses.

A power analysis was performed, in which the ideal sample size for our study was calculated focusing on the diagnostic accuracy of pepsin tests in the previous studies. Precisely, the anticipated SE of the tests was set at 85.0, indicating an expectation of high true positive rate, while the anticipated SP was set at 40.0, acknowledging a relatively high rate of false positives. We assumed an imbalance in the distribution of healthy to diseased individuals, with a ratio (R) set to 1/5, reflecting the prevalence of LPR in the population (≅ 10%). The statistical power, a measure of the study's ability to detect a true effect, was set at a standard value of 0.80. The significance level, a threshold for determining statistical significance, was set at 0.05. Based on these assumptions, a simplified R function was used to calculate the required sample size.

The sensitivity, specificity, positive (PPV) and negative predictive value (NPV) of the pepsin measurement were evaluated considering several thresholds (≥ 16, ≥ 36, ≥ 45, ≥ 75 and ≥ 100 ng/mL). Associations between morning, post-lunch, post-dinner pepsin saliva measurements and clinical findings was investigated through multivariate analysis. The association was defined as low (r_s_ < 0.30), moderate (r_s_ = 0.30–0.60) or strong (r_s_ > 0.60), respectively. The consistency between pepsin measurements, HEMII-pH and clinical findings was assessed with kappa-Cohen analysis.

## Results

### Setting

Forty-two patients and 21 asymptomatic individuals collected saliva samples. The mean age of patients was 48.0 ± 19.6 years. There were 21 females (50%). Twenty-eight patients underwent GI endoscopy, which was unremarkable in 13 (46.0%) patients (Table 1). The LPR diagnosis was positive at the HEMII-pH in 39 patients (92.9%), which was not significantly consistent with the pepsin measurements (Table 2). The mean RSS-12 was 71.1 ± 41.1. The mean RSA was 22.9 ± 10.9. The combination of RSS-12 > 11 with RSA > 14 was significantly consistent with the LPR diagnostic at the HEMII-pH (Table 2).

**Table 1:** Characteristics of patients

Characteristics	
Age (mean, SD)	50.0 ± 16.6
BMI (mean, SD)	26.2 ± 5.5
Male (N, %)	21 (50)
Female (N, %)	21 (50)
*Gastrointestinal endoscopy* (N = 28)	
Normal	13 (46)
Esophagitis	3 (11)
Hiatal hernia	8 (29)
LES insufficiency	9 (32)
Gastritis	7 (25)
*HEMII-pH (mean, SD)*	
Pharyngeal acid events	17.0 ± 17.7
Pharyngeal nonacid events	16.8 ± 22.3
Pharyngeal events (total number)	33.6 ± 27.8
*Saliva pepsin measurements (mean, SD)*	
Morning pepsin test	85.5 ± 96.5
Post-lunch pepsin test	120.2 ± 132.2
Post-dinner pepsin test	104.7 ± 118.6
Mean concentration of pepsin tests	99.5 ± 86.4
Highest concentration of pepsin test	170.0 ± 136.4

BMI, body mass index; HEMII-pH, hypopharyngeal–esophageal multichannel intraluminal impedance-pH monitoring; LES, lower esophageal sphincter; N, number; SD, standard deviation

**Table 2:** Consistency findings

Outcomes	HEMII-pH	
	Kappa	*p* value
Morning pepsin test	0.036	NS
Post-lunch pepsin test	0.024	NS
Post-dinner pepsin test	0.022	NS
Highest pepsin test	0.095	NS
RSS-12 > 11	0.050	NS
RSS-12 > 11 and RSA > 14	0.638	0.005

HEMII-pH, hypopharyngeal–esophageal multichannel intraluminal impedance-pH monitoring; NS, non-significant; RSA, reflux sign assessment; RSS-12, reflux symptom score-12

### Accuracy of pepsin test

The detection of LPR at the pepsin measurements regarding several cutoffs was reported in Table 3. The sensitivity, specificity, PPV and NPV of pepsin measurements was calculated considering a single, two or three measurements (Table 4). The selection of the highest pepsin saliva concentration of the 3 pepsin measurements was associated with the highest accuracy for detecting LPR (70.5%; cutoff ≥ 16 ng/mL). Considering the association of two pepsin measurements, the combination of morning and post-lunch pepsin measurements was 85.7% accurate (Table 4).

**Table 3:** Accuracy of saliva pepsin test according to thresholds

Pepsin tests	Rate
Morning	
≥ 16 ng/mL	65.6
≥ 36 ng/mL	64.5
≥ 45 ng/mL	64.4
≥ 75 ng/mL	62.7
≥ 100 ng/mL	62.7
*Post-lunch*	
≥ 16 ng/mL	62.3
≥ 36 ng/mL	65.6
≥ 45 ng/mL	63.9
≥ 75 ng/mL	60.7
≥ 100 ng/mL	60.7
*Post-dinner*	
≥ 16 ng/mL	61.9
≥ 36 ng/mL	63.5
≥ 45 ng/mL	61.9
≥ 75 ng/mL	61.9
≥ 100 ng/mL	61.9

The best accuracy value was found for fasting saliva pepsin test with a cutoff of ≥ 16 ng/mL

**Table 4:** Accuracy of saliva pepsin test association

Highest sample concentration	
cutoff ≥ 16 ng/mL	Rate
Morning + post-lunch peptest	65.6
Morning + post-dinner peptest	65.6
Post-lunch + post-dinner	62.3
Morning + post-lunch + post-dinner	70.5

The association of the three saliva pepsin tests (morning, post-lunch and post-dinner) reported the best accuracy rate

HEMII-pH, hypopharyngeal–esophageal multichannel intraluminal impedance-pH monitoring

### Sensitivity, specificity and predictive values of pepsin test

The sensitivity, specificity, PPV and NPV of pepsin measurements at cutoffs ≥ 16, ≥ 36, ≥ 45, ≥ 75 and ≥ 100 ng/mL were reported in Table 5. Morning saliva pepsin measurement was 64.9% sensitive and 66.7% specific at cutoffs ≥ 16 ng/mL. At cutoffs ≥ 36 ng/mL, the morning pepsin test was 54.1% sensitive and 80.0% specific. Morning pepsin measurement reported overall higher sensitivity, specificity, PPV and NPV than post-lunch and post-dinner measurements (Table 5). The sensitivity, specificity, PPV and NPV of the ‘highest pepsin measurement’ were 73.0% (95%IC: 69.7, 76.3), 66.7% (95%IC: 66.3, 67.1), 78.9% (95%IC: 78.1, 79.6), and 64.0% (95%IC: 61.4, 66.6) at cutoff ≥ 16 ng/mL, respectively.

**Table 5:** Characteristics of patients according to the reflux profiles

	Morning pepsin test				Post-lunch pepsin test				Post-dinner pepsin test			
	SE	SP	PPV	NPV	SE	SP	PPV	NPV	SE	SP	PPV	NPV
≥ 16 ng/mL	64.9	66.7	75.0	55.2	59.5	66.7	73.3	51.6	59.0	66.7	74.2	50.0
≥ 36 ng/mL	54.1	80.0	80.0	54.0	56.8	79.2	80.8	54.3	53.9	79.2	80.0	51.4
≥ 45 ng/mL	51.4	83.3	81.8	54.1	54.1	79.2	80.0	52.8	51.3	79.2	80.0	50.0
≥ 75 ng/mL	48.6	83.3	81.0	52.6	45.9	83.3	80.9	50.0	48.7	83.3	82.6	50.0
≥ 100 ng/mL	45.7	87.5	84.2	52.5	45.9	83.3	80.9	50.0	48.7	83.3	82.6	50.0

SE, sensitivity; SP, specificity; PPV, positive predictive value; NPV, negative predictive value

### Multivariate analyses

There was a strong association between the number of pharyngeal reflux events and the RSA (r_s_ = 0.634; *p* = 0.006). The level of morning saliva pepsin was moderately associated with the level of post-dinner pepsin (r_s_ = 0.429; *p* = 0.007) and the RSA (r_s_ = 0.578; *p* = 0.019). The post-lunch pepsin level was moderately correlated with the post-dinner pepsin level (r_s_ = 0.369; *p* = 0.019). There was no significant association between the morning and post-dinner pepsin saliva concentrations.

## Discussion

The pepsin saliva test was developed to detect laryngopharyngeal reflux disease without the need for HEMII-pH [6]. To date, studies reported controversial results about the most appropriate time of saliva collection, and the related accuracy, sensitivity, specificity, and predictive values of pepsin saliva measurements. The accuracy and predictive values of the pepsin test were investigated in few studies, which reported controversial results (Table 6) [2, 6, 7, 9, 10, 14–18]. Overall, SE and SP ranged from 29.4 to 100% according to studies, where authors collected saliva sample in the morning, post-meals or after symptoms. Pepsin test appears to be sensitive but not specific. However, most authors included only LPR patients and the lack of control groups may significantly influence the assessment of predictive values and accuracy of pepsin test.

**Table 6:** Literature studies

Authors	Ref	Years	LPR/CT	LPR diagnosis	Sample time	Pepsin analysis	Thresholds	SE	SP	PPV	NPV	Accuracy
Wang	10	2022	112/26	HEMII-pH	From morning	Rapid pepsin	> 45 ng/mL	38.4	84.6	91.5	24.2	55.7
China				≥ 1 HRE	07:00:00 to	lateral flow	Fasting test					
					6:00 PM	Highest saliva	> 45 ng/mL	86.6	80.8	95.1	58.3	73.9
						concentration	Highest test					
Zelenik	9	2021	45/1	HEMII-pH	Morning	PepTest®	≥ 16 ng/mL	48.0	27.0	63.0	40.0	48.0
Czech				> 1 HRE								
Zhang	7	2020	26/4	HEMII-pH	Morning	PepTest®	≥ 16 ng/mL	76.9	25.0	87.0	14.3	87.0
Australia				≥ 2 HRE	1-h post-lunch		≥ 75 ng/mL	57.7	75.0	93.8	21.4	N.P
				≥ 6 PRE	1-h post-dinner							
					When symptoms							
Klimara	2	2019	19/7	HEMII-pH	Morning	ELISA	> 1 ng/mL	29.4	50.0	62.5	20.0	42.0
U.S.A				> 1 HRE	1-h post-lunch	Western blot						
				> 40 PRE	1-h post-dinner	Higesth pepsin						
					1-h post-breakfast	concentration						
Weitzendorfer	15	2019	41/29	Restech	3 samples	PepTest®	> 16 ng/mL	85.4	27.6	62.5	57.1	85.4
Austria				Ryan score		Highest of	> 50 ng/mL	78.1	41.4	65.3	57.1	
				(> 9.4)		3 samples	> 100 ng/mL	68.3	58.6	70.0	56.7	
							> 150 ng/mL	53.7	69.0	71.0	51.3	
							> 216 ng/mL	41.5	86.2	81.0	51.0	
Hayat	6	2015	111/100	MII-pH	Morning	PepTest®	> 16 ng/mL	77.6	63.2	58.4	80.4	N.P
U.K				EAT > 4.2%	1-h post-lunch		> 50 ng/mL	67.2	76.3	67.2	76.8	
					1-h post-dinner		> 100 ng/mL	51.7	74.5	54.5	72.3	
							> 150 ng/mL	41.4	90.8	75.0	69.9	
							> 210 ng/mL	44.2	96.3	95.7	36.5	
Ocak	16	2015	18/2	Dual-probe	N.P	PepTest®	≥ 16 ng/mL	33.0	100	100	14.2	N.P
Turkey				pH testing								
				EAT > 4.2%								
Saritas	17	2012	22/25	Wireless	N.P	PepTest®	≥ 50 ng/mL	50.0	92.0	85.0	68.0	N.P
U.S.A				pH testing								
				EAT > 4.2%								
Potluri	18	2003	3/13	Dual-probe pH testing	When symptoms	Pepsin assay	> 1 ng/mL	100	92.3	N.P	N.P	N.P
U.S.A				≥ 1 proximal acid								
				esophageal event								

EAT, esophageal acid exposure time; (HE)MII-pH, hypopharyngeal–esophageal multichannel-intraluminal impedance-pH monitoring; HRE, hypopharyngeal reflux event(s); LPR/CT, laryngopharyngeal reflux/controls; NP, not provided; NPV, negative predictive value; PPV, positive predictive value; PRE, proximal esophageal reflux event(s); ref., reference; SE, sensitivity; SP, specificity

Our results suggested a variability of the pepsin saliva concentration throughout the day, and the lack of significant consistency with the HEMII-pH results. The morning saliva pepsin measurement appeared to be associated with the highest sensitivity and accuracy, when compared to other measurements. In 2016, Na et al. observed that the average pepsin level upon waking was higher than that measured at any other time [19]. Wang et al. corroborated these findings in a recent study where the morning saliva pepsin measurement was associated with the highest LPR detection rate [10]. The importance of the morning saliva collection was however not supported by Weitzendorfer et al. who observed higher saliva pepsin concentrations after the dinner and the lunch compared to waking concentrations [15]. In other studies, authors reported a variability between morning, post-lunch and post-dinner pepsin saliva concentrations [6, 7] without determining the most adequate time of saliva collection.

The problem of the variability of saliva pepsin concentration and the related discrepancies across studies in accuracy, sensitivity and predictive values may be tackled by the collection of multiple saliva samples. Indeed, as suggested in the present study, recent studies supported that sensitivity, specificity and predictive values may be raised when considering the highest pepsin measurement of 2 or 3 saliva sample collections within the testing day [2, 10, 15]. Considering the highest saliva pepsin measurement, sensitivity, specificity, and PPV found in the present study corroborated those of the literature (Table 6) [2, 10, 15]. Precisely, Wang et al. [10] reported that 55.7% of the true positive cases were missed by considering a single pepsin test. Similarly, Hayat et al. and Zhang et al. supported that the accuracy, sensitivity, specificity, and predictive values of pepsin saliva measurements were improved when considering the highest pepsin saliva concentration of three or four measurements, respectively [6, 7].

To date, the variability of pepsin saliva concentration throughout the day is not fully understood. Several factors may influence the gastric pepsin secretions, the esophageal motility, the relaxation of sphincters, and the related pepsin saliva concentration. First, it has been suggested that the foods and beverages consumed during the testing day may influence the pepsin saliva concentration [16, 17]. On the one hand, foods and beverages may influence the gastric secretion of pepsin, and, therefore, the pepsin concentration into the stomach content that may refluxate into the upper aerodigestive tract tissues [18, 19]. On the other hand, acid, spicy, low-protein, and high-fat foods may increase the number of transient relaxations of esophageal sphincters, leading to an increased number of pharyngeal reflux events that contain pepsin [16, 17]. Regarding the influence of diet, the differences across studies from different world regions should be interpreted according to the diet habits of populations.

Both esophageal sphincter tonicity and motility are known to be influenced by the autonomic nerve function [20, 21]. The activation of sympathetic nervous system may impair the esophageal antireflux barriers (sphincter tonicity and esophageal motility), leading to pharyngeal reflux events. In that way, patients with stress, anxiety or depressive findings at the time of the diagnosis/testing should have higher number of pharyngeal reflux events and, theoretically, higher pepsin saliva concentration compared to patients without autonomic nerve dysfunction [20, 21]. In addition to these factors, it is important to keep in mind that the saliva pepsin measurements highlight the extracellular pepsin concentrations, while recent studies suggested a potential internalization of pepsin into the Golgi apparatus of pharyngeal cells [22], which makes undetectable a part of refluxate pepsin.

To the best of our knowledge, the present report is the first study investigating accuracy, sensitivity, specificity and predictive values of pepsin saliva measurements according to the time of saliva collection. The primary limitation of the present study was the homogeneity of the study population, which mainly included patients with a positive diagnostic at the HEMII-pH and only 21 asymptomatic individuals. The lack of healthy individuals benefiting from HEMII-pH may be considered as a limitation but HEMII-pH is costly and inconvenience for asymptomatic patients. Future studies are needed to better understand the low SE and SP of pepsin test, and to investigate the presence of other gastroduodenal enzymes in the saliva of patients. Indeed, the presence of other enzymes, such as bile salts, should explain the mucosa injuries and related symptoms and findings without detected pepsin.

## Conclusion

The collection of several saliva pepsin samples improves the detection rate of LPR. The consideration of the highest concentration of multiple saliva pepsin collections was associated with the highest detection rate, and sensitivity. In case of high clinical LPR suspicion and negative pepsin test, a HEMII-pH study could provide further diagnostic information. Future studies are needed to confirm the most adequate number and time of saliva sample collection for the pepsin measurement.

## Data Availability

Data are available on request.

## Abbreviations

GI: Gastrointestinal
HEMII-pH: Hypopharyngeal–esophageal multichannel intraluminal impedance-pH monitoring
LPR: Laryngopharyngeal reflux
N/PPV: Negative/positive predictive value
RSA: Reflux sign assessment
RSS-12: Reflux symptom score-12
